# Hybrid Ablation in Atrial Fibrillation: Bridging Mechanistic Understanding and Clinical Practice

**DOI:** 10.3390/jcdd12080313

**Published:** 2025-08-19

**Authors:** Flavia Ravelli, Stefano Branzoli, Alessandro Cristoforetti, Silvia Quintarelli, Alessio Coser, Paolo Moggio, Mark La Meir, Carlo de Asmundis, Luigi Pannone, Francesco Onorati, Roberto Bonmassari, Massimiliano Marini

**Affiliations:** 1 Laboratory of Biophysics and Translational Cardiology, Department of Cellular, Computational and Integrative Biology (CIBIO), Centre for Medical Sciences (CISMed), University of Trento, 38123 Trento, Italy; alessandro.cristoforetti@unitn.it; 2 Department of Cardiac Surgery, S. Chiara Hospital, APSS PA of Trento, 38122 Trento, Italy; stefano.branzoli@apss.tn.it (S.B.); francesco.onorati@unitn.it (F.O.); 3 Cardiac Surgery Department, Vrije Universiteit Brussel, Universitair Ziekenhuis Brussel, 1050 Brussels, Belgium; mark.lameir@uzbrussel.be; 4 Department of Cardiology, S. Chiara Hospital, APSS PA of Trento, 38122 Trento, Italy; silvia.quintarelli@apss.tn.it (S.Q.); alessio.coser@apss.tn.it (A.C.); paolo.moggio@apss.tn.it (P.M.); roberto.bonmassari@apss.tn.it (R.B.); massimiliano.marini@apss.tn.it (M.M.); 5 Heart Rhythm Management Centre, Postgraduate Program in Cardiac Electrophysiology and Pacing, Vrije Universiteit Brussel, Universitair Ziekenhuis Brussel, 1050 Brussels, Belgium; carlo.deasmundis@uzbrussel.be (C.d.A.); luigi.pannone@uzbrussel.be (L.P.)

**Keywords:** cardiac arrhythmias, atrial fibrillation, atrial fibrillation ablation, hybrid ablation, atrial fibrillation mechanisms, atrial remodeling

## Abstract

Atrial fibrillation (AF), the most prevalent sustained cardiac arrhythmia, poses a significant burden on global morbidity and healthcare expenditure. Although endocardial catheter ablation and surgical ablation are established therapeutic strategies, each exhibits inherent limitations in achieving comprehensive substrate modification. Hybrid ablation therapy, integrating both endocardial and epicardial approaches, aims to overcome these limitations by enabling the more extensive and transmural targeting of arrhythmogenic foci and the complex atrial substrate. This review synthesizes the electrophysiological basis and mechanistic rationale underpinning hybrid AF ablation, highlighting its potential for an enhanced efficacy compared to isolated techniques. Furthermore, it introduces the emerging paradigm of three-dimensional ablation within this evolving field.

## 1. Introduction

Atrial fibrillation (AF) is a complex cardiac arrhythmia arising from the intricate interplay of multiple pathophysiological mechanisms spanning diverse spatial and temporal scales. The multifactorial nature of AF, encompassing macroscopic electrophysiological and clinical features alongside microscopic genetic and molecular factors, creates a highly heterogeneous substrate for both triggers and perpetuating arrhythmic activity [1,2,3]. Despite this growing understanding, the effective targeting of these interwoven mechanisms to achieve durable therapeutic outcomes remains a significant challenge [4,5].

Conventional therapies, including antiarrhythmic drugs and catheter ablation, demonstrate variable success rates, particularly in the challenging patient population with persistent AF. In a recent meta-analysis of 11 studies that compared the efficacy and safety of cryoballoon (CB) and radiofrequency (RF) ablation in the treatment of persistent AF, the incidence of freedom from atrial tachyarrhythmia events was 62.3% and 61.9%, respectively, at a minimum follow-up of 12 months. Nine studies reported the occurrence of total complications between the CB group and the RF group, and there was no statistical difference between the two groups (CB 0.6% vs. RF 0.5%, *p* = 0.68) [6]. While catheter ablation primarily targets endocardial pulmonary vein isolation (PVI), surgical ablation offers the potential for more extensive lesion sets, but is associated with increased procedural risks. Hybrid ablation therapy has emerged as a strategy to capitalize on the strengths of both endocardial and epicardial approaches, aiming to achieve more durable lesions and reduce the incidence of arrhythmia recurrence [7,8]. The mechanistic rationale for this synergy lies in the ability of combined endocardial and epicardial lesion sets to create a more complete conduction block, especially at critical isthmuses and within fibrotic regions that often exhibit resistance to endocardial catheter-based energy delivery.

The evolution of the hybrid ablation concept directly addresses the recognized limitations of standalone procedures in achieving sustained success, particularly in persistent AF. Multiple studies have documented the limited efficacy of catheter ablation monotherapy in this population, with the reported recurrence rates exceeding 44.1% at 8 years in persistent AF and 57.3% in long-standing persistent AF [9]. In contrast, isolated or hybrid thoracoscopic ablation have demonstrated improved outcomes compared to catheter ablation [10]. Notably, a recent systematic review suggests that hybrid thoracoscopic ablation offers an enhanced efficacy compared to isolated thoracoscopic ablation without a significant increase in complications [11].

The clinical evidence supporting the benefits of hybrid ablation has strengthened considerably in the past five years with the publication of three randomized controlled trials (one single-center and two multicenter) [12,13,14]. Across these trials, hybrid ablation consistently demonstrated a superior efficacy in maintaining the sinus rhythm in patients with persistent AF compared to catheter ablation alone. The landmark CONVERGE trial, a multicenter RCT, established the superiority of the hybrid approach (thoracoscopic surgical convergent technique followed by catheter ablation) over standard endocardial catheter ablation, with a success rate at one year of 67.7% versus 50.0%, respectively. There were no deaths, cardiac perforations, or atrioesophageal fistulas in the trial, with a total of 2.9% of the patients in the hybrid group with primary safety events within seven days [12]. This finding was further corroborated by the single-center HARTCAP-AF trial, which also demonstrated the superiority of hybrid ablation (thoracoscopic surgical clamp technique followed by catheter ablation) over a catheter-only strategy [13]. After a 12-month period, the freedom of atrial tachyarrhythmias was 89% in the hybrid group compared to 41% in the catheter ablation group. There were no deaths, strokes, need for pacemaker implantation, or conversions to sternotomy, and the incidence of adverse events was similar between the groups (21% vs. 14%) [13]. Finally, in 2023, Doll et al. published the multicenter CEASE-AF randomized trial [14]. In this study, patients with drug-refractory, symptomatic persistent, or long-standing persistent AF were assigned in a 2:1 ratio to either a staged hybrid ablation or catheter ablation with a potential repeat ablation. The hybrid ablation procedure included thoracoscopic radiofrequency (RF) ablation and left atrial appendage exclusion with second-stage endocardial catheter ablation performed 3–6 months later. The primary endpoint was found to be significantly higher in the hybrid group than in the catheter ablation group (71.6 vs. 39.2%, *p* < 0.001), as was the major complications (7.8% vs. 5.8%, *p* = 0.75) throughout the 30 days after the index procedures.

The compelling results from these trials have prompted a significant shift in the recent 2024 ESC AF guidelines, upgrading the recommendation for hybrid ablation in patients with symptomatic, antiarrhythmic, drug-refractory, persistent AF from Class IIb of the previous ESC AF guidelines [15] to Class IIa, with a Level of Evidence A of the latest ESC AF guidelines [16].

## 2. Basic Mechanisms Underlying Atrial Fibrillation

**Figure 1:**
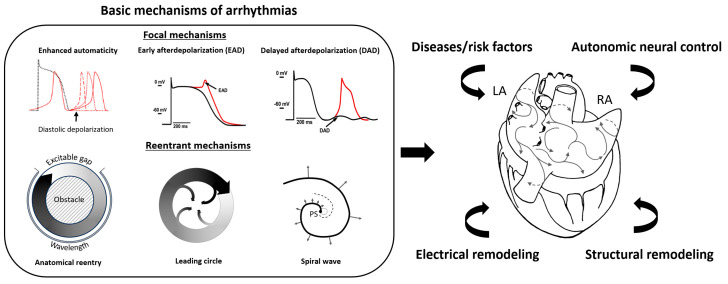
Pathophysiological mechanisms underlying AF. The **left** panel illustrates the basic mechanisms of arrhythmias that can initiate and interfere with the propagation process of multiple wavelets, as depicted in the **right** panel. The resulting complex fibrillatory pattern can be further modulated by underlying comorbidities, autonomic neural control, and diverse forms of tissue remodeling. See text for details.

The genesis and perpetuation of atrial fibrillation (AF) are supported by a complex interplay of diverse electrophysiological mechanisms, culminating in a self-sustaining arrhythmic milieu [1,2]. Characterized as a “complete arrhythmia,” AF can be initiated and maintained by the convergence of several factors. Primary driver mechanisms encompass abnormal automaticity and early and delayed afterdepolarizations, alongside various manifestations of re-entrant activity [16], including re-entry around anatomical obstacles, leading circle re-entry, and the dynamic propagation of spiral waves (Figure 1, left panel). These high-frequency sources orchestrate the chaotic and irregular fibrillatory conduction observed throughout the atrial myocardium. The intricate pathogenesis of AF is further modulated by tissue remodeling processes, neural control mechanisms, and underlying cardiac and systemic pathologies, all of which contribute to the evolving arrhythmogenic substrate (Figure 1, right panel) [17,18,19].

A fundamental aspect of AF pathogenesis is the presence of focal triggers, with the pulmonary veins (PVs) representing the most recognized sources of ectopic electrical activity [20]. However, as AF progresses to a persistent stage, non-PV triggers gain increasing significance [21,22]. Ectopic sources in the posterior left atrial wall, the ligament of Marshall, and structures such as the left atrial appendage (LAA) contribute to AF maintenance, undermining the success rate of conventional pulmonary vein isolation strategies. Beyond focal triggers, the multiple wavelet hypothesis offers another key mechanism for AF sustenance [23]. This posits that the arrhythmia is perpetuated by the continuous and disorganized propagation of multiple re-entrant wavelets throughout the atrial myocardium, leading to spatiotemporal heterogeneity in electrical activation, disrupting coordinated atrial contraction, and reinforcing the fibrillatory state. Recent insights underscore the role of rotors and localized re-entrant drivers in AF maintenance [24]. Stable high-frequency micro-re-entries may sustain AF by continuously generating rapid electrical activity, which, in turn, drives the chaotic propagation of impulses throughout the atria. Another crucial element of AF pathophysiology is the growing recognition that this arrhythmia is not purely two-dimensional, but involves a three-dimensional electrical and structural substrate [25]. The complex layered architecture of the atrial myocardium creates significant dissociation between endocardial and epicardial activation patterns [26]. During AF, this endo-epicardial asynchrony contributes to the perpetuation of re-entrant circuits, reinforcing the fibrillatory state and complicating ablation strategies. This phenomenon is particularly pronounced in persistent AF, where extensive electrical and structural remodeling results in a heightened conduction heterogeneity, facilitating sustained fibrillatory activity within a three-dimensional framework.

### 2.1. The Triple Remodeling Driving Atrial Fibrillation Progression

The transition from paroxysmal to persistent AF is fundamentally orchestrated by a process of atrial remodeling, which progressively and profoundly alters the electrophysiological, contractile, and structural architecture of the atria [17]. These evolving perturbations establish an arrhythmic milieu for AF perpetuation, rendering the arrhythmia progressively refractory to termination.

A feature of electrical remodeling in persistent AF is the abbreviation of the atrial effective refractory period, a direct consequence of sustained high-frequency activation. This rapid depolarization of atrial myocytes reduces the action potential duration, thereby facilitating wavebreak genesis, a pivotal electrophysiological derangement promoting re-entrant circuits and sustaining AF. Furthermore, the physiological rate adaptation of atrial refractoriness becomes compromised. Another critical determinant of electrical remodeling is conduction velocity heterogeneity. Persistent AF favors inhomogeneous conduction slowing, which is largely attributable to altered ion channel expression and atrial fibrosis [27,28]. These alterations disrupt uniform impulse propagation, increasing the propensity for wavefront fractionation and re-entrant circuit formation that perpetuate the arrhythmia. Moreover, growing evidence underscores the salient role of endo-epicardial electrical dissociation in the intricate complexity of AF [29,30]. Normally, the atrial endocardium and epicardium exhibit synchronized activation; however, in AF, this coordination is lost, culminating in asynchronous activation. The resultant electrical dissociation generates a more complex, disorganized substrate, promoting continuous fibrillatory conduction and rendering AF more resistant to conventional therapies.

Concomitant with electrical remodeling, persistent AF is also characterized by substantial structural derangements within the atrial myocardium. Several AF-induced structural changes in atrial myocytes have been observed, including an increase in the cell size, the perinuclear accumulation of glycogen, myolysis, changes in the mitochondrial shape and size, the fragmentation of the sarcoplasmic reticulum, the dispersion of nuclear chromatin, and changes in the quantity and localization of structural cellular proteins [31]. A component of this remodeling cascade is atrial stretch and dilatation, serving as both an etiological factor and a downstream consequence [32,33]. Acute atrial stretch, by opening stretch-activated channels, shortens the refractory period, increases the dispersion of atrial refractoriness, slows the conduction velocity, and increases the vulnerability to AF [34]. Chronic atrial dilatation, whether due to underlying functional or structural heart diseases or extracardiac factors, is a common feature of cardiovascular and non-cardiovascular risk factors predisposing to AF [35,36,37]. Chronic stretch causes heterogeneous changes in the atrial architecture, such as myocyte hypertrophy, dedifferentiation, apoptosis, the loss of contractile elements, and fibrosis [38], thereby contributing to the arrhythmia’s self-perpetuation. An increased atrial dilatation in a genetic form of atrial dilated cardiomyopathy is also known to dramatically increase the fibrotic tissue in the atria, further contributing to the emergence of atrial arrhythmias [39]. Persistent AF exhibits a robust association with augmented fibrotic tissue deposition, disrupting normal electrical conduction pathways [25]. Fibrosis generates conduction block, promoting re-entry, further stabilizing the arrhythmia, and diminishing the likelihood of successful rhythm control with conventional ablation strategies [40,41]. Additionally, gap junction dysfunction assumes a pivotal role in AF maintenance [42,43]. Connexins, notably Cx40 and Cx43, are indispensable for intercellular electrical coupling and synchronized activation. In AF, aberrant connexin expression and distribution affect impulse propagation, contributing to conduction heterogeneity and increasing the substrate for re-entry [44,45]. Collectively, these electrical and structural modifications generate a progressively more arrhythmogenic atrial environment, reinforcing AF persistence and diminishing the efficacy of standard therapeutic interventions [33,46].

### 2.2. Autonomic Nervous System Involvement in Atrial Fibrillation

The autonomic nervous system (ANS) exerts a pivotal and multifaceted influence on both the initiation and perpetuation of atrial fibrillation [47]. Through the intricate and integrated actions of its sympathetic and parasympathetic branches, the ANS dynamically shapes the atrial electrophysiological landscape, promoting arrhythmogenesis and facilitating the progression from paroxysmal to the more refractory persistent form of AF [18].

Sympathetic overactivity, often a sequela of underlying pathologies such as myocardial ischemia, heart failure, hypertension, and sleep apnea, is a significant contributor to both the initiation and perpetuation of atrial fibrillation through several mechanisms. Elevated sympathetic activity profoundly impacts atrial electrophysiology and structure by promoting focal ectopic firing through enhanced automaticity, early or delayed afterdepolarizations, and contributing to adverse structural remodeling. Parasympathetic activity also significantly modulates atrial fibrillation pathogenesis. Its acute augmentation profoundly shortens the atrial effective refractory period and increases spatial dispersion of repolarization, thus critically shortening the wavelength of re-entrant excitation and fostering a vulnerable substrate for re-entrant circuits. Thus, elevated vagal activity promotes functional or anatomical wavebreak, thereby critically contributing to AF maintenance and perpetuation.

Beyond these direct sympathetic and parasympathetic effects, the intrinsic cardiac autonomic nervous system (ICANS), particularly the ganglionated plexi (GP) nestled within the epicardial fat pads, functions as a crucial modulator of AF initiation and persistence [48]. These GP harbor clusters of autonomic neurons that engage in complex interactions with both sympathetic and parasympathetic afferents, establishing intricate regulatory feedback loops that influence atrial excitability [49]. Evidence from animal models and human studies indicates that an autonomic imbalance within these plexi can predispose to a heightened electrical instability, further reinforcing the maintenance of AF [48,50]. Notably, recent findings have also demonstrated a role for ICANS plasticity in shaping the atrial substrate in human AF [51].

In concert, these autonomic mechanisms contribute to a highly dynamic and heterogeneous atrial milieu, wherein derangements in a sympathetic and parasympathetic tone can both trigger and sustain AF episodes. This intricate interplay underscores the paramount importance of targeting autonomic modulation, particularly in cases of persistent AF, where conventional ablation techniques alone may prove insufficient for achieving enduring rhythm control.

## 3. Mechanistic Insights into Persistent Atrial Fibrillation and Implications for Ablation

**Figure 2:**
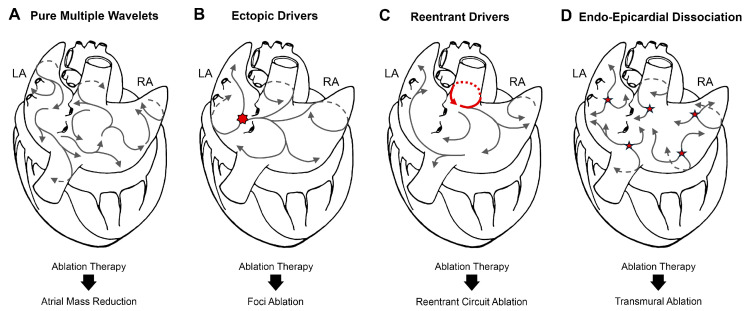
Mechanisms of AF and targeted ablative therapy. Panel (**A**) illustrates a fibrillation process sustained by a purely multiple wavelet mechanism. Ablation therapy should aim to reduce the number of wavelets perpetuating the arrhythmia. In this scenario, a maze ablation procedure, designed to reduce the excitable atrial mass, may represent the elective ablation therapy. Panel (**B**) depicts a fibrillation episode sustained by one or more ectopic foci (red star). Targeted foci ablation is the elective therapy. Panel (**C**) describes a “mother wave–daughter waves” mechanism, wherein secondary wavelets are perpetuated by a re-entrant circuit (red circle). Under these circumstances, therapy should be directed at terminating the re-entrant activity of the mother wave. Panel (**D**) portrays the complexity of the three-dimensional fibrillatory propagation pattern, highlighting endo-epicardial dissociation and the presence of multiple epicardial breakthroughs (indicated by stars). Intramural ablation lesions are consequently necessary in this scenario. LA: left atrium, RA: right atrium.

In contrast to paroxysmal atrial fibrillation (AF), often driven by focal pulmonary vein (PV) triggers amenable to pulmonary vein isolation (PVI), persistent AF is sustained by a complex, substrate-dependent arrhythmogenic milieu [22,30]. The intricate interplay of electrical and structural remodeling fosters a self-sustaining environment that renders conventional ablation strategies suboptimal. Consequently, effective management in most cases requires a comprehensive approach extending beyond stand-alone PVI, targeting the different mechanisms that can coexist in the specific patient (Figure 2).

A hallmark of persistent AF is the heterogeneous atrial substrate characterized by extensive fibrosis, fatty infiltration, and conduction abnormalities [52]. These structural derangements create zones of slow conduction and block, facilitating the formation and perpetuation of re-entrant circuits, including intramural pathways [41]. Even in the absence of initiating triggers, the remodeled atrial tissue sustains the arrhythmia, underscoring substrate modification as a critical therapeutic target in persistent AF ablation [53]. This aligns with scenarios where a purely multiple wavelet mechanism sustains fibrillation, necessitating procedures like maze ablation to reduce the excitable atrial mass, as illustrated in Figure 2A.

As AF chronicity increases, the significance of non-PV triggers escalates. While paroxysmal AF frequently originates from ectopic foci within the PVs, persistent AF is often maintained by additional triggers arising from regions such as the posterior left atrium, the ligament of Marshall, and the left atrial appendage. These non-PV triggers contribute substantially to AF recurrence post-PVI, emphasizing the necessity of their identification and targeted ablation [22], as described for ectopic foci ablation in Figure 2B, to improve the long-term outcomes. Furthermore, persistent AF is characterized by the emergence of endo-epicardial electrical dissociation and complex three-dimensional re-entrant circuits [54]. Recent evidence has demonstrated that atrial fibrosis in these patients extensively penetrates the atrial walls, forming a three-dimensional substrate for intramural re-entries [25]. This electrical uncoupling between endocardial and epicardial layers leads to asynchronous activation, promoting stable three-dimensional re-entrant circuits that are challenging to eliminate with standard endocardial ablation [55,56]. This understanding highlights the need for advanced mapping and ablation techniques that account for transmural conduction patterns [57], often necessitating the creation of intramural lesions to address the complex fibrillatory propagation patterns and multiple epicardial breakthroughs (Figure 2D). Moreover, some persistent AF cases involve a “mother wave–daughter waves” mechanism, where therapy must be directed at terminating the re-entrant activity of the mother wave, another complex scenario requiring targeted intervention (Figure 2C).

Compounding these challenges is the progressive nature of remodeling, which reinforces AF persistence over time. A prolonged arrhythmia duration is correlated with more extensive electrical and structural alterations, further stabilizing the fibrillatory state. This self-perpetuating cycle underscores the importance of early intervention to mitigate irreversible atrial remodeling. Delayed treatment diminishes the likelihood of successful rhythm control and necessitates more extensive ablation strategies targeting both triggers and the underlying substrate.

Collectively, these mechanistic insights underscore the imperative for a tailored and comprehensive approach to persistent AF ablation. While PVI remains a foundational element, addressing the arrhythmogenic substrate (e.g., via maze-like approaches), targeting non-PV triggers or local re-entries, and acknowledging the three-dimensional complexity of conduction abnormalities (e.g., requiring intramural lesions for intramural re-entries and endo-epicardial dissociation) are crucial steps toward enhancing the long-term success rates and reducing AF recurrence.

## 4. Mechanistic Basis of Hybrid Ablation

**Figure 3:**
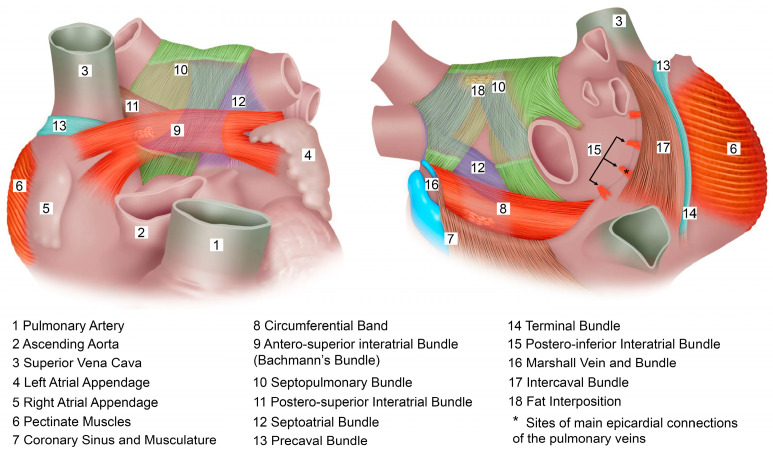
Architecture of atrial musculature. **Left**: atrial muscular bundles from anterior view. **Right**: atrial muscular bundles from posterior view. See text for details.

The electrophysiological rationale for hybrid ablation lies in the complementary nature of endocardial and epicardial lesion formation. Surgical ablation, the first successful invasive treatment for atrial fibrillation (AF), was pioneered by James Cox in 1987, culminating in the refined “Cox-maze procedure” [58,59]. While effective, the Cox-maze procedure’s limitations, primarily its open-chest approach and associated higher complication rates, led to the development of catheter ablation as a less invasive endocardial treatment. However, a critical challenge for catheter ablation remains the consistent creation of transmural lesions. The complex architecture of the atrial musculature, as illustrated in Figure 3, features a smooth-walled left atrium overlaid by muscular bundles with varying orientations.

Notably, Bachmann’s bundle connects the right and left atria across the interatrial groove, while the septopulmonary and septoatrial bundles originate from the septum. The septoatrial bundle predominantly covers the left atrial body, whereas the septopulmonary bundle encircles the pulmonary veins. Along the roof and part of the posterior wall, these two septal bundles overlap, but are separated by a layer of fat [60]. By integrating both endocardial and epicardial ablation strategies, hybrid ablation overcomes the limitations inherent in standalone catheter-based techniques and is less invasive in comparison to traditional surgical techniques, effectively achieving three-dimensional ablation.

A key advantage of epicardial surgical ablation is its capacity to create durable, transmural lesions and access regions beyond the pulmonary veins, including the left atrial posterior wall, the left atrial appendage, the ligament of Marshall, and the septopulmonary bundle—a recognized contributor to AF maintenance due to its electrical insulation and propensity for wavelet re-entry. Conversely, endocardial catheter ablation offers a real-time electrophysiological assessment, enabling the confirmation of conduction block and the identification of gaps within the surgical lesions. Beyond validating lesion durability, the endocardial approach allows for the targeted ablation of residual endocardial triggers, including non-pulmonary vein (non-PV) ectopic foci, which become increasingly significant as AF progresses. These non-PV triggers, often originating from structures such as the coronary sinus, the superior vena cava, and the mitral or tricuspid annulus, play a crucial role in sustaining the arrhythmia. Sometimes an extra endocardial ablation is necessary after the hybrid approach ablation for the treatment of residual atrial flutter [16].

The synergy between surgical and catheter-based techniques proves particularly advantageous in persistent and long-standing AF, where progressive structural remodeling creates a highly arrhythmogenic substrate. This mechanistic understanding underscores the importance of a tailored, substrate-modifying approach in the management of advanced AF, highlighting hybrid ablation as a strategy for improving the outcomes in patients with complex, treatment-resistant forms of the arrhythmia.

### Future Directions

Future research should prioritize the development of energy delivery systems capable of reliably achieving transmural lesions with an enhanced safety and efficiency [61]. A transformative advancement would be the advent of mapping technologies enabling the real-time, simultaneous acquisition of integrated endocardial and epicardial electroanatomic data, ultimately yielding a truly three-dimensional electrophysiological model of the atria.

## 5. Conclusions

Hybrid ablation therapy represents a significant evolution in AF treatment, strategically leveraging the complementary strengths of endocardial and epicardial approaches to enhance the procedural efficacy. A thorough understanding of its mechanistic rationale is paramount for optimizing patient selection and procedural strategies. Further rigorous studies are essential to refine techniques and establish standardized protocols to maximize the clinical outcomes and define its role in the AF treatment paradigm.

## Data Availability

Not applicable.

## Abbreviations

The following abbreviations are used in this manuscript:

AF	Atrial fibrillation
PVI	Pulmonary vein isolation
RCT	Randomized controlled trial
RF	Radiofrequency
PVs	Pulmonary veins
CB	Cryoballoon
LAA	Left atrial appendage
ANS	Autonomic nervous system
ICANS	Intrinsic cardiac autonomic nervous system
GP	Ganglionated plexi
RA	Right atrium
LA	Left atrium
